# Discussion of Tumors by Electricity

**Published:** 1875-05-15

**Authors:** 


### DISCUSSION OF TUMORS BY ELECTRICITY.

*From the Clinic*, *May* 1, 1875.

IN the discussion of tumors, this agent may be employed in two or three ways, but always with the continuous current. It is most effective when the mass is benign, as in pelvic cellulitis, cystic or orchitic enlargements of the testicles or in goitre—these affections having all been successfully treated by electricity. Where the tumor is of a malignant nature, long continued treatment is frequently as efficacious as any form of surgical interference, and may be thoroughly relied upon to arrest development. In these cases, however, there is scarcely anything known, and time may or may not demonstrate the value of electricity over the methods of treatment now in vogue.

Within the past three months, several fibroids have been submitted to treatment by galvanism, with highly encouraging results. I have long been doubtful of the necessity of introducing needles into such tumors, and attempting dissolution of the mass by powerful galvanic currents employed in this way, since the great density of the tissues precludes more than a small portion thereof being affected at once, and since, in my hands at least, abscesses have formed between the needles as the sole effect of such treatment. Two fibroids have been treated ineffectually by galvano-puncture, which afterward yielded to and were removed by the process I am about to refer to, which may be called external electrolysis. One other, which was transferred to my charge seven weeks since by Dr. Clarence T. Gardner, an old anæmic uterine interstitial, almost as hard as bone, has, in that time, been reduced more than one-third by actual measurement, and the patient otherwise improved in health. The mode of application in this case will serve to illustrate the process usually adopted. An uterine electrode, carefully insulated up to within one and a half inches of a bulbous point, is introduced into the uterus as far as possible, and connected with the negative pole of an intensity galvanic battery. The positive pole is attached to a sponge-covered disk, which is placed under the sacrum, and a current strong enough to produce a distinct pricking sensation, passed between them for at least thirty minutes, daily. When the negative pole is withdrawn from the cavity of the uterus, it is always thickly coated with oxide, which is harmless, the metal employed being iron. The result is slow chemical decomposition of the fibroid and energetic stimulation of the absorbents, whereby the mass is gradually renewed. It is true that the process is long and slow; but, in the great majority of cases, patients prefer this to the more rapid action of the needles, even were such action effectual, which I doubt. In the case referred to, no other operative interference is possible, save the hypodermic use of ergotin; and even the most enthusiastic supporters of that plan of treatment, confess its barrenness of result, when the tumor is not partially, at least, myomatous. In fact, the employment of galvano-puncture in solid masses like old fibroids, or indeed, in enlargements of any kind, where the texture of tissue is extremely dense, is to be avoided as worse than useless, and as capable of doing great harm. If any good can be attained at all by the action of galvanism in these tumors, it will be best attained, I believe, by the plan just described, which is absolutely harmless, painless, and usually beneficial to general health.

It is only where the substance of the mass is partly or wholly fluid, that galvano-puncture is permissible, and where its effects are so brilliant in curative result as published records show. In hard tumors, where time is an object, I am of opinion that the knife operation is better than any plan yet proposed; but to many, especially to delicate women of strong nervous temperament, the idea of cutting is so repugnant, that they are willing to submit to external electrolysis for any length of time, provided a favorable result may finally be reached; and this has been done in at least three cases.—*The Present Status of Electricity:* Wm. F. Hutchinson, M.D.

